# Miniaturized Rapid Electrochemical Immunosensor Based on Screen Printed Carbon Electrodes for *Mycobacterium tuberculosis* Detection

**DOI:** 10.3390/bios13060589

**Published:** 2023-05-29

**Authors:** Noura Zouaghi, Shahid Aziz, Imran Shah, Ahmed Aamouche, Dong-won Jung, Brahim Lakssir, El Mostafa Ressami

**Affiliations:** 1 LISA Laboratory, National Applied Science School, Cadi Ayyad University, Marrakech 40000, Morocco; n.zouaghi@mascir.ma (N.Z.); a.aamouche@uca.ma (A.A.); 2 Moroccan Foundation for Advanced Science, Innovation and Research, Digitalization & Microelectronics Smart Devices Laboratory, Rabat Design Center, Rabat 10112, Morocco; b.lakssir@mascir.ma (B.L.); m.ressami@mascir.ma (E.M.R.); 3 Department of Mechanical Engineering, Jeju National University, 102 Jejudaehak-ro, Jeju-Si 63243, Republic of Korea; shahid@jejunu.ac.kr; 4 Institute of Basic Sciences, Jeju National University, 102 Jejudaehak-ro, Jeju-Si 63243, Republic of Korea; 5 Department of Aerospace Engineering, College of Aeronautical Engineering, National University of Sciences and Technology, Risalpur 24090, Pakistan; imranshahswabi@gmail.com; 6 Faculty of Applied Energy System, Major of Mechanical Engineering, Jeju National University, 102 Jejudaehak-ro, Jeju-Si 63243, Republic of Korea

**Keywords:** *Mycobacterium tuberculosis*, LAMP-Electrochemical, carbon screen-printed electrode, CV analysis

## Abstract

In 2019, over 21% of an estimated 10 million new tuberculosis (TB) patients were either not diagnosed at all or diagnosed without being reported to public health authorities. It is therefore critical to develop newer and more rapid and effective point-of-care diagnostic tools to combat the global TB epidemic. PCR-based diagnostic methods such as Xpert MTB/RIF are quicker than conventional techniques, but their applicability is restricted by the need for specialized laboratory equipment and the substantial cost of scaling-up in low- and middle-income countries where the burden of TB is high. Meanwhile, loop-mediated isothermal amplification (LAMP) amplifies nucleic acids under isothermal conditions with a high efficiency, helps in the early detection and identification of infectious diseases, and can be performed without the need for sophisticated thermocycling equipment. In the present study, the LAMP assay was integrated with screen-printed carbon electrodes and a commercial potentiostat for real time cyclic voltammetry analysis (named as the LAMP-Electrochemical (EC) assay). The LAMP-EC assay was found to be highly specific to TB-causing bacteria and capable of detecting even a single copy of the *Mycobacterium tuberculosis* (Mtb) IS*6110* DNA sequence. Overall, the LAMP-EC test developed and evaluated in the present study shows promise to become a cost-effective tool for rapid and effective diagnosis of TB.

## 1. Introduction

Tuberculosis (TB), caused by *Mycobacterium tuberculosis*, a communicable disease, was alone responsible for the highest mortality rate due to a single infectious microbe until the start of the COVID-19 pandemic [1,2]. Nearly 10 million people are estimated to have developed TB globally in 2019 alone, with more than 1.4 million deaths [3]. However, over 21% of these patients were either not diagnosed at all or diagnosed but not reported to public health authorities [3]. Concerningly, the World Health Organization (WHO) reported in 2020 that if the number of TB patients that are diagnosed or detected by authorities decreases by 50% in a 3-month period, the number of TB deaths could increase by 400,000 [3,4]. 

In 2014, the WHO adopted the ‘End TB’ strategy, with the primary targets of this strategy being to reduce TB incidence rate, mortality, and economic burden on patients and their families [2]. Quantitatively, the WHO’s targets for the ‘End TB’ strategy are to reduce TB incidence rate by 80% and TB mortality by 90% by the year 2030 [2]. This is a highly ambitious target, especially since the lockdowns imposed to combat the COVID-19 pandemic have negatively impacted the healthcare of TB patients, delaying both diagnosis and treatment [5].

Conventionally, TB is diagnosed using the tuberculin skin test and sputum smear microscopy. However, the tuberculin test has a wide-ranging sensitivity (68–95%), which varies depending on the concentration and potency of the tuberculin used [6]. Moreover, this test takes up to 72 h for diagnosis [6]. While sputum smear microscopy is a cost-effective and logistically feasible method, it also has the disadvantage of low sensitivity (up to 60%) [7,8].

In order to accomplish the WHO’s ‘End TB’ goals, it is therefore critical to develop newer and more effective tools to combat the global TB epidemic, including new vaccine(s), shorter treatment regimens, and importantly, rapid and effective point-of-care (POC) diagnostic tests [7,9]. The Xpert MTB/RIF assay, introduced in 2010 and endorsed by the WHO, offers one such POC method for simultaneous detection of MTB and its sensitivity towards the primary TB treatment drug rifampin (RIF) [10]. This real time polymerase chain reaction (PCR)-based assay, conducted on sputum samples, takes less than 2 h and has been reported to have a sensitivity of 88% and a specificity of 98% [11,12]. However, the sensitivity of PCR-based detection is lower (67%) for the detection of intestinal TB when biopsy (tissue) samples are used [13]. Importantly, the applicability of this method is restricted by the need for specialized laboratory equipment and the substantial cost of scaling-up in low and middle-income countries where the burden of TB is high [10]. Another method, called loop-mediated isothermal amplification (LAMP) amplifies nucleic acids under isothermal conditions and helps in the early detection and identification of infectious diseases, including TB and COVID-19 [14,15]. The isothermal nature (typical temperature range 60–65 °C) of the LAMP reaction allows it to be performed without the need for sophisticated thermocycling equipment, unlike PCR [14,15]. The LAMP method has a higher amplification efficiency, successfully detecting even a few target amplicons in the reaction mixture [15]. Overall, LAMP offers several advantages such as ease of use, rapid amplification and detection, and cost-effectiveness [15]. In 2016, the WHO approved the use of TB-LAMP as both a substitute and a follow-up test for microscopy-based TB diagnosis in symptomatic patients [16].

Notably, the LAMP reaction produces a white magnesium pyrophosphate residue in the reaction mixture, which allows for users to determine the qualitative outcome of the reaction simply through the naked eye [17,18]. However, the sensitivity of the naked eye measurement is low since different observers may report the outcome differently, leading to erroneous diagnoses [18]. The use of a sensor to quantify the analytes (such as the amount of by-product) produced during the LAMP reaction can improve the sensitivity of this technique.

Electrochemical sensors, based on redox reactions, often allow the simultaneous quantification of multiple analytes in a reaction. The presence of analytes and the occurrence of multiple redox reactions at the surface of a working electrode of the electrochemical sensors generate a potential difference and result in a flow of current across the system [19,20,21]. The measured electrical parameters are functions of analyte concentration.

In redox-based sensors, an electrochemical technique called cyclic voltammetry (CV) can be used to measure the response rate of an active redox solution through a potential sweep between multiple set potential values [22]. CV typically uses a sensor system consisting of three electrodes, i.e., a working electrode (W.E.), a reference electrode (R.E.), and a counter electrode (C.E.). A potentiostat is incorporated into the system to control the potential applied at the working electrode [22].

In the present study, the outcome of the LAMP assay has been quantified using screen-printed carbon electrodes and a commercial potentiostat for real time cyclic voltammetry analysis (LAMP-Electrochemical (EC) assay), thereby allowing a rapid and effective detection of Mtb.

## 2. Materials and Methods

### 2.1. Instrumentation

**Figure 1:**
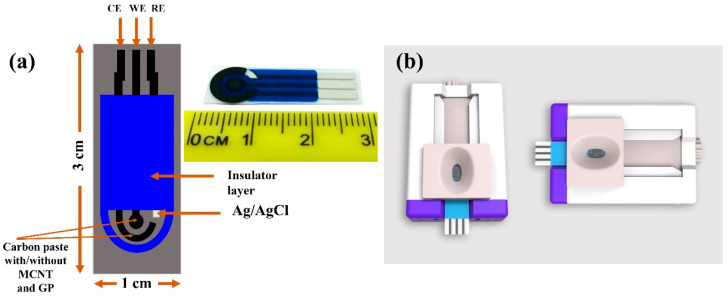
SPCE structure. (**a**) The SPCEs had a three-electrode configuration, with a CE, a WE, and an RE. The distances between the three electrodes were determined based on the structure of the socket of potentiostat connectors. (**b**) The SPCE socket.

The screen-printed carbon electrodes (SPCEs) utilized in this work (PalmSens B.V., Randhoeve, The Netherlands) possessed a conventional three-electrode configuration constructed on a corundum ceramic base of the dimensions 25.4 mm × 7.26 mm, with a carbon working electrode (WE), a silver reference electrode (RE), and a carbon counter electrode, which acted as the auxiliary electrode (Figure 1). CV measurements were obtained at constant room temperature with the help of a potentiostat (PalmSens3™, PalmSens B.V., Randhoeve, The Netherlands), which was operated through the PSTrace software (version 5.8), as described elsewhere [23].

### 2.2. Constructing the Recombinant Plasmid

PCR was used to amplify the full-length Mtb-IS6110 gene. Specifically, the forward primer IS-F (IS-FOP) and the backward primer IS-R (IS-BOP) were used to form amplicons of a 202 bp fragment of the gene. The obtained amplified sequence was ligated into the pGEM^®^-T Easy Vector (Promega Corporation, Madison, WI, USA) as described in the manufacturer’s protocol. PCR was further used to identify positive clones upon transformation of competent Escherichia coli DH5α cells (New England Biolabs, Ipswich, MA, USA). The PCR amplicons thus obtained were identified at their respective expected sizes (base-pair lengths). Subsequently, the Wizard^®^ SV Minipreps DNA Purification System (Promega Corporation, Madison, WI, USA) was used to purify the plasmid DNA, which was then serially diluted (1:4 ratio) to prepare stocks [24]. Finally, the LAMP reaction was conducted using 5 µL of the template, unless otherwise stated.

### 2.3. Optimization of the LAMP Reaction

**Figure 2:**
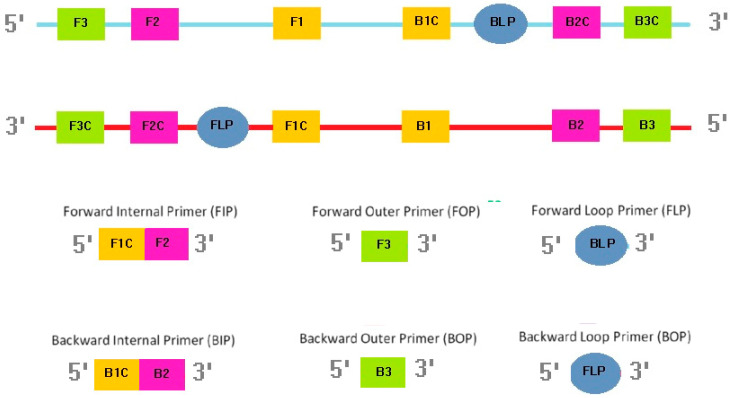
Location and sequence of primers.

All LAMP primers (outer, inner, and loop) were designed based on previously described sequences of the Mtb-IS6110 gene (GenBank accession number: X17348) (Figure 2, Table S1). The LAMP-specific primer design software PrimerExplorer V5 (Eiken Chemical Company Ltd., Tokyo, Japan) was used to design the primers listed in Table S1. The LAMP assay reaction mixture (25 µL) contained IS-Forward Internal Primer (FIP) and IS-Backward Internal Primer (BIP) (1.6 μM), loop primers (IS-Forward Loop Primer (FLP) and IS-Backward Loop Primer (BLP)) (0.8 μM), IS-Forward Outer Primer (FOP) (F3) and IS-Backward Outer Primer (BOP) (B3) (0.2 μM), deoxynucleoside triphosphates (dNTPs; 2 mM), betaine (0.8 M; Sigma-Aldrich, St. Louis, MO, USA), Tris-HCl (20 mM; pH 8.8), potassium chloride (KCl; 10 mM), ammonium sulfate ((NH4)2SO4; 10 mM), magnesium sulfate (MgSO4; 9 mM), Triton X-100 (0.1% *v*/*v*), Bst 2.0 WarmStart DNA Polymerase (8 U; New England Biolabs, Ipswich, MA, USA), and the DNA template (5 μL) [24]. Agarose gel (1% w/v) electrophoresis was used to determine the optimal reaction temperature and time. The positive control was the genomic DNA of Mtb H37Ra reference strain (ATCC, Manassas, VA, USA), while the negative control was diethyl pyrocarbonate (DEPC)-treated water.

### 2.4. Specificity of the LAMP Reaction

DNA templates (100 ng each) prepared from nineteen known bacterial species, including two tuberculous mycobacterial species, three non-tuberculous mycobacterial species and fourteen non-mycobacterial species, were used. Specifically, the specificity of the LAMP-EC assay was evaluated using the 19 bacterial strains listed in Table S2. As indicated in Table S2, the method identified only two species—Mtb and *M. bovis*—as positive for Mtb. Indeed, 12 mycobacterial species, together known as the *Mycobacterium tuberculosis* Complex (MTBC), are capable of causing tuberculosis infection in several animal species [25]. As many as 8 of these, including *M. bovis*, are capable of spreading the disease in humans too [25,26,27]. Notably, the LAMP-EC assay results were negative for the remaining 17 bacterial strains used in the present study. These results suggest that the LAMP-EC assay is highly specific to MTBC.

### 2.5. Comparing the Sensitivities of LAMP-EC and Single Step (SS)-LAMP

Serial dilutions of the Mtb-IS6110 recombinant plasmid DNA were prepared ($10^{6}$, $10^{5}$, $10^{4}$, $10^{3},\;10^{2},$ 10, and 1 copy per reaction) to perform a comparative evaluation of the molecular sensitivities (or limits of detection) of the LAMP-EC method and an in-house SS-LAMP assay [24]. The in-house SS-LAMP assay was conducted at 65 °C for 60 min, as described previously [24].

### 2.6. Electrochemical Measurements of LAMP Amplicons

**Figure 3:**
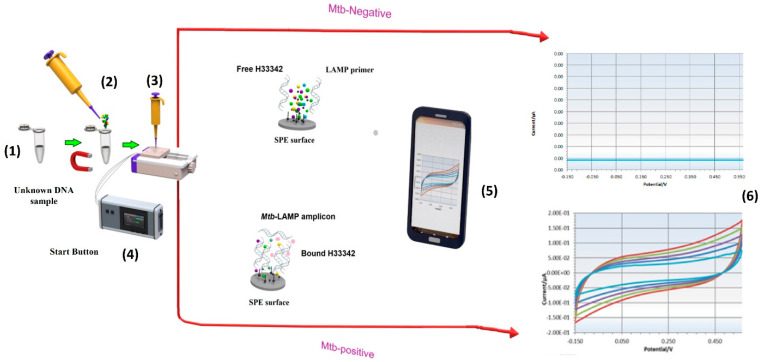
Schematic of the LAMP-EC method. Palmsens software. (**1**): LAMP incubation at 65 °C. (**2**): add H33342 redox probe. (**3**): Drop LAMP product-H33342 mixture onto Screen-printed carbon electrodes (SPCE) inserted into socket and connected whit potentiostat. (**4**): press Start bottom. (**5**): The result appears on PS Trace software in Smart Phone or Laptop. (**6**): real time cyclic voltammetry analysis (conducted at room temperature) allowing a rapid and effective detection of Mtb.

Hoechst 33342 (H33342) molecules were used as redox probes in the LAMP-EC assay. These molecules serve as redox-active indicators [28]. As the DNA-loaded electrode is exposed to H33342, the redox-active dye molecules bind to the adenine-thymine (AT)-rich regions in the DNA [29]. This binding occurs both through hydrogen bonding and electrostatic interactions arising between the positively charged N-methylpiperazine group in the H33342 molecules and the negatively charged DNA backbone [30]. The electrostatic interactions affect the potential and current, which can be recorded via cyclic voltammetry [31]. To electrochemically detect the amplicons synthesized by the LAMP assay, oxidation signals, generated by the binding of the DNA amplicons with H33342 molecules, were measured using CV in the potential range of −0.15 V to 0.57 V with a scan rate of 100 mV/s (Figure 3). A potentiostat was used to control the potential in all electrochemical experiments. Data from these experiments were analyzed using the PSTrace 5.8 software package.

### 2.7. Electrochemical Sensing Experiments

To remove any contamination from the electrode surface, the SPCEs were cleaned by applying 50 µL of 0.1 M Prussian Blue solution (pH 7) on the sensing area, as reported previously [32]. Only the electrodes that generated identical voltammograms were utilized in the subsequent experiments to ensure reproducibility.

## 3. Results and Discussion

### 3.1. Scanning Electrone Microscope (SEM) Analysis

**Figure 4:**
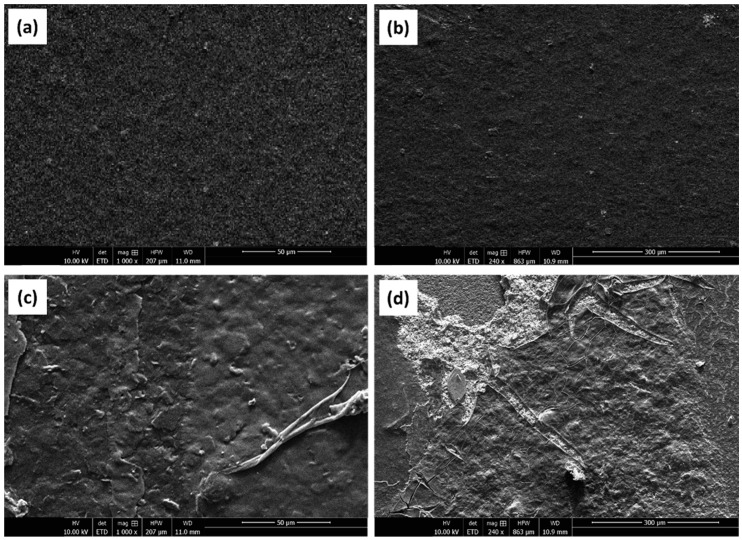
SEM images of (**a**,**b**) a bare SPCE, (**c**,**d**) SPCE whit LAMP reaction.

Figure 4 shows the SEM images of the SPCEs at different stages of preparation. The surface of the LAMP-functionalized SPCE differed in morphology in comparison to the bare electrode. Specifically, a thread-like arrangement of spherical grains could be observed among the carbon particles. The thread-like structures were found to be dense with some porosity, which made the surface of the LAMP-functionalized SPCE more uniform than that of the bare SPCE.

### 3.2. Electrochemical Characterization

**Figure 5:**
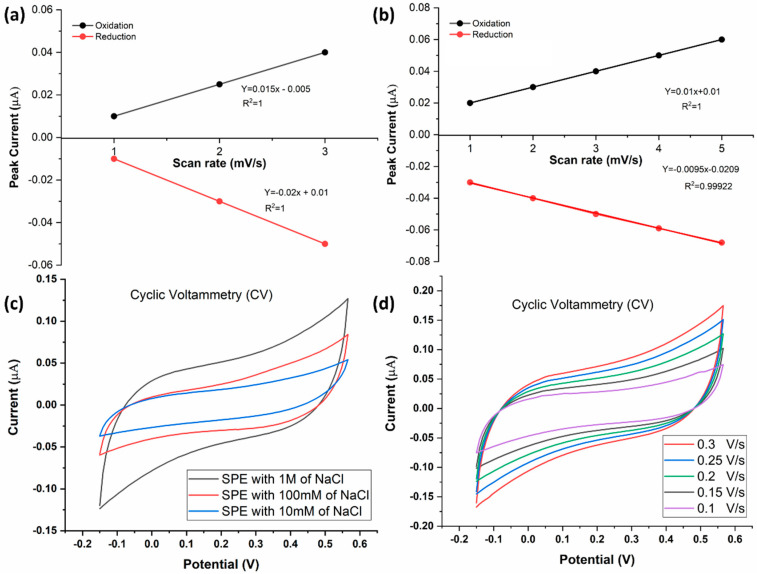
(**a**) Standard curve showing the relationship between the anodic/cathodic current of NaCl and NaCl concentration). (**b**) Standard curve showing the relationship between the anodic/cathodic current (µA) and the scan rate1/2 ((mV/S)1/2). (**c**) CV at different concentrations of NaCl solution: 10 mM, 100 mM, and 1 M. Voltage was swept from −0.15 V to +0.57 V and back. (**d**) Voltammograms of a 1 M NaCl, generated using different scan rates (0.1–0.3 V/s).

To characterize the functionality of the electrode, CV was first performed using sodium chloride (NaCl) as the electrolyte (Figure 5). The electrode exhibited expected behavior, registering an increase in peak current with increasing NaCl concentration (Figure 5a,c). The electrode was further characterized by conducting CV at different scan rates (0.10 V/s, 0.15 V/s, 0.20 V/s, 0.25 V/s, and 0.30 V/s) at a fixed NaCl concentration of 1 M (Figure 5b,d). The peak current measurements expectedly increased with the scan rate, suggesting normal electrode behavior (Figure 5b,d).

**Figure 6:**
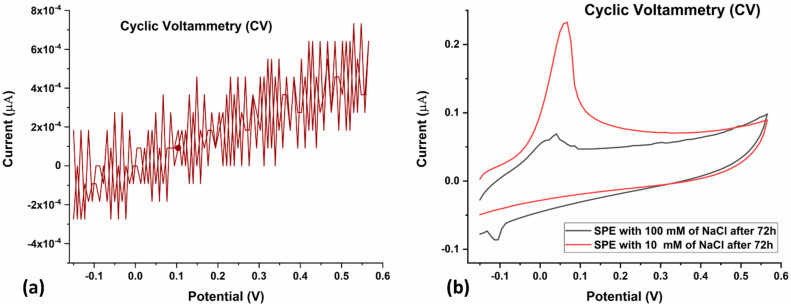
(**a**) CV of bare SPE after 72 h of utilization. Voltage was swept from −0.15 V to +0.57 V and back, at a scan rate of 0.10 V/s. (**b**) CV of 10 mM (red) and 100 mM (grey) NaCl after 72 h of utilization of the SPE. Voltage was swept from −0.15 V to +0.57 V and back, at a scan rate of 0.10 V/s.

To assess the reusability of the SPCE, the electrode was subjected to CV again after 72 h of utilization (Figure 6a,b). The positive dependence of peak electrode current on electrolyte concentration was maintained, which indicates that the SPCE is reusable even after 72 h of utilization for CV (Figure 6b). 

**Figure 7:**
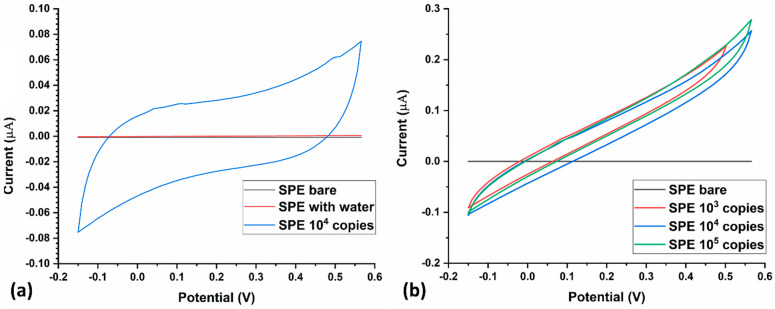
(**a**) Current response of the LAMP-EC assay using negative (bare, water) and positive (10^4^ copies) Mtb samples; (**b**) Variation in current response of the LAMP-EC assay using different Serial dilutions of the Mtb-IS6110 recombinant plasmid DNA (10^5^, 10^4^, 10^3^).

After electrochemical characterization of the LAMP-EC electrode using NaCl, the setup was tested with H33342 dye as the redox probe, with or without the Mtb samples. The LAMP-EC assay resulted in a clear distinction between negative and positive Mtb samples, as shown in Figure 7a. Further, as expected, the peak current during CV experiments increased with the concentration of the Mtb DNA amplicons (Figure 7b). 

**Figure 8:**
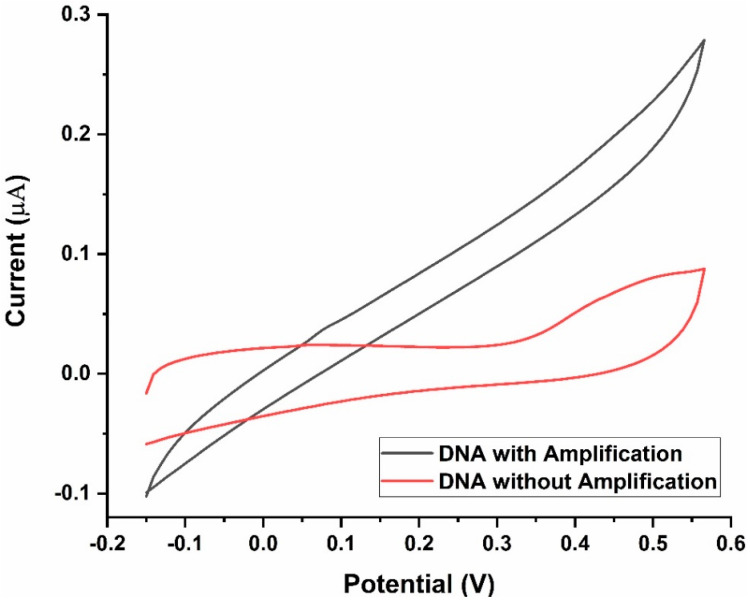
Current response of the LAMP-EC assay Before and after DNA amplification.

The need for conducting the LAMP reaction before CV measurements was clear from the differences in the voltammograms of samples with and without amplification (Figure 8). Mtb-positive samples without LAMP-mediated amplification of DNA had a significantly lower peak current (nearly three-fold lower) than those with amplification.

### 3.3. Effect of Scan Rate

**Figure 9:**
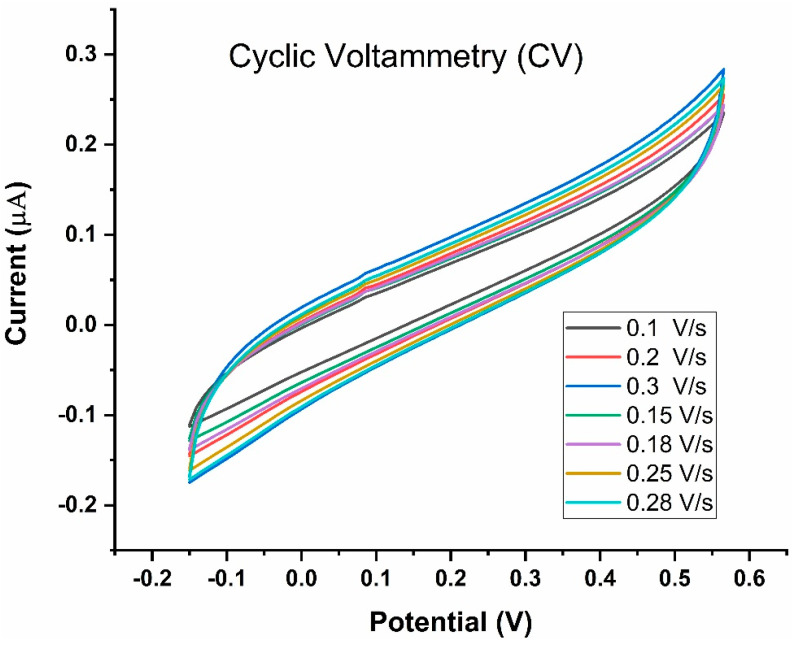
Cyclic Voltammetry of the LAMP-EC assay using positive Mtb sample conducted at different scan rates (20–250 mV/s).

To evaluate the dynamic nature of the SPCEs developed in the present study, LAMP reaction was performed at various scan rates. Increasing the scan rate from 0.10 V/s to 0.30 V/s resulted in an anticipated increase in peak current [22], as shown in Figure 9, suggesting that SPCE functionality was maintained in the presence of the H33342 redox probe. The scan rate of 0.1 V/s was identified for subsequent experiments as the standard curve between the anodic current of analytes included this scan rate. Further, higher scan rates (>0.1 V/s) would mean greater peak potentials of redox species. A suitable scan rate would be one that generates larger current values and smaller peak potentials.

### 3.4. Repeatability and Reproducibility Assessment

**Figure 10:**
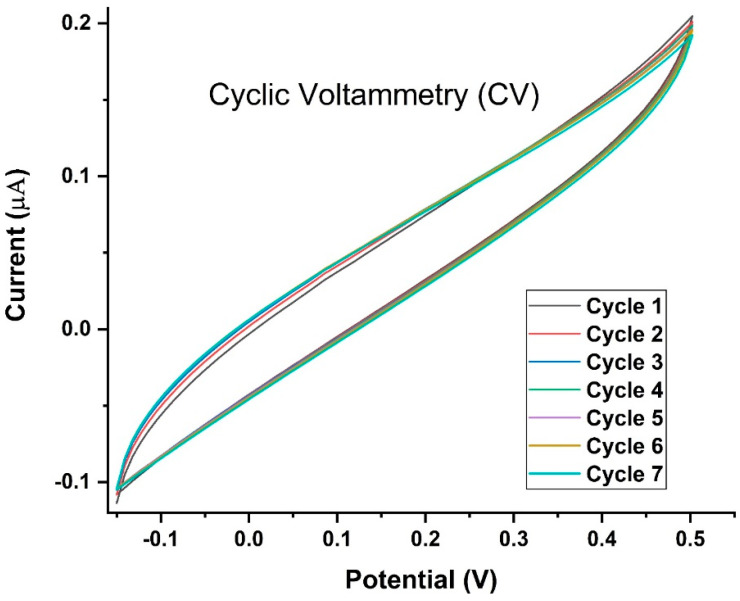
Cyclic Voltammetry for nine cycles of the LAMP-EC assay using positive Mtb sample. Voltage was swept from −0.15 V to +0.57 V and back, at a scan rate of 0.10 V/s.

The repeatability of the SPCE-based measurements was assessed by conducting CV for nine consecutive cycles. For up to nine cycles, the electrode constructed in the present study exhibited a stable behavior towards the oxidation of each analyte with a relatively low standard deviation (Figure 10).

The reproducibility of the electrode’s analyte detection capability was assessed using 10 electrodes. The SPCE showed good reproducibility with similar cyclic voltammograms generated by all tested electrodes for the same DNA concentration.

### 3.5. Optimization of LAMP Conditions

To determine the optimum reaction temperature, the LAMP assay was conducted for a duration of 60 min at three different temperatures (60 °C, 65 °C, and 68 °C) using the extracted DNA as a template (Figure S1). The amplification results were evaluated using standard gel electrophoresis [24]. The LAMP assay conducted at 60 °C yielded no DNA bands, similar to the negative control, which indicates that LAMP primers were not active at this temperature. Meanwhile, the LAMP reaction carried out at 65 °C resulted in strong DNA bands. However, at 68 °C, the intensity of the DNA bands was lower, suggesting that the LAMP primers had become less effective at this temperature. From these observations, 65 °C was selected as the optimal temperature for conducting the LAMP assay.

To identify the optimum reaction time for the LAMP assay that could maximize the number of amplicons synthesized at 65 °C, the reaction was conducted for four durations of time: 30 min, 45 min, and 60 min. As can be noted from the agarose gel electrophoresis images in Figure, the intensity of the DNA bands increased from 30 min to 45 min, suggesting that a higher number of LAMP amplicons are synthesized in 45 min. However, the band intensity did not increase substantially from 45 min to 60 min, suggesting that the LAMP reaction is optimally completed in 45 min.

### 3.6. Comparing the Sensitivities of the LAMP-LFD, SS-LAMP, and LAMP-EC Methods

In the previous section, it has been established that the LAMP method developed in this study had an analytical sensitivity of one copy/reaction for Mtb plasmid detection. A Loop-Mediated Isothermal Amplification Combined with a Lateral Flow Dipstick LFD-LAMP, developed in a previous study, was used for comparison [33]. This study shows $10^{4}$ as a limit of detection of the IS66100 sequence (Figure S2b). Therefore, there was an in-house SS-LAMP assay (conducted through a LAMP Lab-on-Card device) [24]. This single-step assay does not require DNA extraction and denaturation and enzyme inactivation steps [24]. The limit of detection of the SS-LAMP assay was previously found to be 10 copies (Figure S2c) of the IS6110 sequence [24]. In the present study, the limit of detection of the LAMP-EC assay was found to be as low as one copy of the IS6110 sequence (Figure S2a), while agarose gel electrophoresis of the same samples could not detect fewer than 1000 copies of the DNA sequence (Figure S2c). In addition, since LAMP-EC is a quantitative method, the likelihood of human errors, that can impact the interpretation of turbidity or colorimetric LAMP results by the naked-eye, will be low. Moreover, LAMP-EC is a label-free and low-cost with a high sensitivity.

## 4. Discussion

The growing TB epidemic has necessitated the development of efficient and effective strategies for disease identification, prevention, and treatment. It is important to develop quick diagnostic tests that are also accurate, sensitive, and specific to TB, while being cost-feasible in developing nations. The present study demonstrates the application of one such test, referred to as the LAMP-EC assay. The test is quantifiable through CV and is highly sensitive, specific, and reproducible. Moreover, the SPCE-based device used to conduct the LAMP-EC assay is reusable even after 72 h of utilization.

The LAMP-EC assay described in the present study exhibits high accuracy and high specificity towards Mtb. Similar results have been demonstrated in a recent study [34]. Indeed, LAMP is highly specific (and thus, accurate) because of the use of six different primers that are capable of recognizing eight different regions on the target sequence [35]. This makes DNA amplification highly efficient [35]. A previous study using LAMP for TB diagnosis has reported detection of Actinomycetes in addition to Mtb [36], which makes their method less specific and less accurate than the present method which specifically detected only TB-causing species.

The use of the IS*6110* sequence as a target for a LAMP assay has been reported previously [24,35]. However, the sensitivity of the test reported in the present study is a fold higher (or limit of detection a fold lower) than that reported in a previous LAMP-based TB diagnosis study [24]. The present test is also more sensitive than the LAMP test developed by Iwamoto et al. [36] as well as the commonly used Amplicor test, as the limit of detection for both of these tests was 5–50 copies per reaction. The sensitivity of the present test is, however, comparable to that reported by Aryan et al. [35]. Their study reported limits of detection of 1 and 200 copies of the IS*6110* sequence for LAMP and PCR detection tests, respectively [35]. Notably, the sensitivity of the presented technique is substantially greater than that of a recently reported eosin photopolymerization-based method for colorimetrically quantifying LAMP products, for which the limit of detection was found to be 30 copies per µL [37]. Comparisons with other emerging techniques for TB diagnosis, such as plasmonic fiberoptic absorbance-based biosensors, need to be performed in a more direct manner in future studies, as current units and methods of limit of detection measurements are inconsistent with the ones used in the present study [38].

The aspect that sets the present study apart from other LAMP-based detection methods is the ability to quantify the test results by means of an electrochemical sensor. The use of CV to detect and quantify nucleic acid molecules in electrolytic solutions has been reported in numerous studies [39,40,41]. For example, Asrat et al. [40] reported the use of carbon fiber microelectrodes and fast-scan CV for the detection of *Caenorhabditis elegans* DNA and RNA. Another study reported the use of CV for pre-treatment of gold-leaf electrodes to be used for LAMP-based detection of HIV and HPV amplicons [41]. The use of CV in the present study allows for a highly reproducible and noise-free quantification of the electrical response generated by the interaction between DNA amplicons and the H33342 dye. 

H33342 molecules thus acted as redox probes in the LAMP-EC assay. Other Hoechst dye molecules, such as H33258, have also commonly been used as redox probes in CV-based quantification of microbial nucleic acids [34,42]. Future studies could compare the nature of the cyclic voltammograms generated in the presence of different Hoechst/DNA-binding dyes. Note that NaCl was used in the present study as the electrolyte for characterizing the electrochemical properties of the SPCE. This is because NaCl maintains a high ionic strength of the solution, thereby preventing fluctuations in the electric field [43]. Other salts may also be used and could be tested in future studies, particularly if the interaction between the DNA and salt molecules is to be studied [44,45].

The goal of the present study was to develop an effective TB detection method that may be employed as a POC diagnostic method in resource-poor settings. It is therefore important to conduct further studies on the LAMP-EC assay that will focus on testing patient sputum samples for evaluating its clinical applicability. Subsequently, the method may also be tested for the detection of TB (particularly, intestinal TB) in biopsy/tissue samples [13].

## 5. Conclusions

The present study sought to develop a low-resource and reliable DNA-based tuberculosis diagnosis method by improving upon an isothermal amplification technique. To this end, the LAMP method was integrated with an electrochemical sensor-based DNA detection technique through which TB diagnosis can be performed in resource-poor regions. The developed LAMP-EC assay allows for a rapid and highly sensitive, specific, and reproducible quantitative analysis of MTBC DNA amplicons, making it an adequate, low-resource electrochemical sensor for the diagnosis of tuberculosis infection.

## Data Availability

The data presented in this study are available in the present article and the associated supplementary material.

## Supplementary Materials

The following supporting information can be downloaded at: https://www.mdpi.com/article/10.3390/bios13060589/s1, Figure S1: (a) Optimization of LAMP reaction temperature using 10-fold serially diluted Mtb-DNA as template. The reactions were carried out at different temperatures for 45 min. (b) Optimization of LAMP reaction time at the optimal temperature using various amounts of template; Figure S2: Detection sensitivity data of MTB genomic DNAs at concentration range of 10^−1^ to 10^−6^ dilutions obtained from (a) EC-LAMP, (b) LFD- LAMP, and (c) SS-LAMP. Lanes M and N represent DNA ladder marker and negative control (no-DNA template), respectively; Table S1: Primer and probe sequences for the LAMP-EC biosensor-based assay; Table S2: Specificity of LAMP-EC using the mini-potentiostat.

## References

[1] Wikman-Jorgensen P., López-Velez R., Llenas-García J., Treviño B., Pascual R., Molina I., Domínguez Á., Torrús D., Giardín J.M.R., Monge-Maillo B. (2020). Latent and active tuberculosis infections in migrants and travellers: A retrospective analysis from the Spanish+ REDIVI collaborative network. Travel Med. Infect. Dis..

[2] World Health Organization (2021). Global Tuberculosis Report 2021.

[3] Chakaya J., Khan M., Ntoumi F., Aklillu E., Fatima R., Mwaba P., Kapata N., Mfinanga S., Hasnain S.E., Katoto P.D. (2021). Global Tuberculosis Report 2020—Reflections on the Global TB burden, treatment and prevention efforts. Int. J. Infect. Dis..

[4] World Health Organization (2020). Global Tuberculosis Report 2020.

[5] The Lancet Public Health (2021). Renewing the fight to end tuberculosis. Lancet Public Health.

[6] Monaghan M., Doherty M., Collins J., Kazda J., Quinn P. (1994). The tuberculin test. Vet. Microbiol..

[7] García-Basteiro A.L., DiNardo A., Saavedra B., Silva D.R., Palmero D., Gegia M., Migliori G.B., Duarte R., Mambuque E., Centis R. (2018). Point of care diagnostics for tuberculosis. Pulmonology.

[8] Ng B.Y., Wee E.J., West N.P., Trau M. (2015). Rapid DNA detection of *Mycobacterium tuberculosis*-towards single cell sensitivity in point-of-care diagnosis. Sci. Rep..

[9] Uplekar M., Weil D., Lonnroth K., Jaramillo E., Lienhardt C., Dias H.M., Falzon D., Floyd K., Gargioni G., Getahun H. (2015). WHO’s new end TB strategy. Lancet.

[10] Vassall A., van Kampen S., Sohn H., Michael J.S., John K., den Boon S., Davis J.L., Whitelaw A., Nicol M.P., Gler M.T. (2011). Rapid diagnosis of tuberculosis with the Xpert MTB/RIF assay in high burden countries: A cost-effectiveness analysis. PLoS Med..

[11] Blakemore R., Story E., Helb D., Kop J., Banada P., Owens M.R., Chakravorty S., Jones M., Alland D. (2010). Evaluation of the analytical performance of the Xpert MTB/RIF assay. J. Clin. Microbiol..

[12] Steingart K.R., Sohn H., Schiller I., Kloda L.A., Boehme C.C., Pai M., Dendukuri N. (2013). Xpert^®^ MTB/RIF assay for pulmonary tuberculosis and rifampicin resistance in adults. Cochrane Database Syst. Rev..

[13] Jin X.J., Kim J.M., Kim H.K., Kim L., Choi S.J., Park I.S., Han J.Y., Chu Y.C., Song J.Y., Kwon K.S. (2010). Histopathology and TB-PCR kit analysis in differentiating the diagnosis of intestinal tuberculosis and Crohn’s disease. World J. Gastroenterol..

[14] Augustine R., Hasan A., Das S., Ahmed R., Mori Y., Notomi T., Kevadiya B.D., Thakor A.S. (2020). Loop-mediated isothermal amplification (LAMP): A rapid, sensitive, specific, and cost-effective point-of-care test for coronaviruses in the context of COVID-19 pandemic. Biology.

[15] Parida M., Sannarangaiah S., Dash P.K., Rao P., Morita K. (2008). Loop mediated isothermal amplification (LAMP): A new generation of innovative gene amplification technique; perspectives in clinical diagnosis of infectious diseases. Rev. Med. Virol..

[16] World Health Organization (2016). The Use of Loop-Mediated Isothermal Amplification (TB-LAMP) for the Diagnosis of Pulmonary Tuberculosis: Policy Guidance.

[17] Mori Y., Nagamine K., Tomita N., Notomi T. (2001). Detection of loop-mediated isothermal amplification reaction by turbidity derived from magnesium pyrophosphate formation. Biochem. Biophys. Res. Commun..

[18] Tian B., Ma J., Zardán Gómez de la Torre T., Bálint A., Donolato M., Hansen M.F., Svedlindh P., Stromberg M. (2016). Rapid newcastle disease virus detection based on Loop-mediated isothermal amplification and optomagnetic readout. ACS Sens..

[19] Farooqi H.M.U., Sammantasinghar A., Kausar F., Farooqi M.A., Chethikkattuveli Salih A.R., Hyun K., Choi K.H. (2022). Study of the Anticancer Potential of Plant Extracts Using Liver Tumor Microphysiological System. Life.

[20] Karimi-Maleh H., Arotiba O.A. (2020). Simultaneous determination of cholesterol, ascorbic acid and uric acid as three essential biological compounds at a carbon paste electrode modified with copper oxide decorated reduced graphene oxide nanocomposite and ionic liquid. J. Colloid Interface Sci..

[21] Noura Z., Shah I., Aziz S., Ahmed A., Jung D.W., Brahim L., ElMostafa R. (2022). Wearable healthcare monitoring based on a microfluidic electrochemical integrated device for sensing glucose in natural sweat. Sensors.

[22] Elgrishi N., Rountree K.J., McCarthy B.D., Rountree E.S., Eisenhart T.T., Dempsey J.L. (2018). A practical beginner’s guide to cyclic voltammetry. J. Chem. Educ..

[23] Farooqi H.M.U., Kang B., Khalid M.A.U., Salih A.R.C., Hyun K., Park S.H., Choi K.H. (2021). Real-time monitoring of liver fibrosis through embedded sensors in a microphysiological system. Nano Converg..

[24] Bentaleb E.M., Abid M., El Messaoudi M.D., Lakssir B., Amzazi S., Sefrioui H., Benhassou H.A. (2016). Development and evaluation of an in-house single step loop-mediated isothermal amplification (SS-LAMP) assay for the detection of *Mycobacterium tuberculosis* complex in sputum samples from Moroccan patients. BMC Infect. Dis..

[25] Kanipe C., Palmer M.V. (2020). Mycobacterium bovis and you: A comprehensive look at the bacteria, its similarities to *Mycobacterium tuberculosis*, and its relationship with human disease. Tuberculosis.

[26] Centers for Disease Control and Prevention (2005). Human tuberculosis caused by *Mycobacterium bovis*—New York City, 2001–2004. Morb. Mortal. Wkly. Rep..

[27] Thoen C., LoBue P., De Kantor I. (2006). The importance of *Mycobacterium bovis* as a zoonosis. Vet. Microbiol..

[28] Rashid J.I.A., Yusof N.A. (2017). The strategies of DNA immobilization and hybridization detection mechanism in the construction of electrochemical DNA sensor: A review. Sens. Bio-Sens. Res..

[29] Bucevičius J., Lukinavičius G., Gerasimaitė R. (2018). The use of hoechst dyes for DNA staining and beyond. Chemosensors.

[30] Zhang X.X., Brantley S.L., Corcelli S.A., Tokmakoff A. (2020). DNA minor-groove binder Hoechst 33258 destabilizes base-pairing adjacent to its binding site. Commun. Biol..

[31] Sufen W., Tuzhi P., Yang C.F. (2002). Electrochemical studies for the interaction of DNA with an irreversible redox compound–Hoechst 33258. Electroanal. Int. J. Devoted Fundam. Pract. Asp. Electroanal..

[32] Pasakon P., Mensing J.P., Phokaratkul D., Karuwan C., Lomas T., Wisitsoraat A., Tuantranont A. (2019). A high-performance, disposable screen-printed carbon electrode modified with multi-walled carbon nanotubes/graphene for ultratrace level electrochemical sensors. J. Appl. Electrochem..

[33] Kaewphinit T., Santiwatanakul S., Chansiri K. (2013). Colorimetric DNA based biosensor combined with loop-mediated isothermal amplification for detection of *mycobacterium tuberculosis* by using gold nanoprobe aggregation. Sens. Transducers.

[34] Jaroenram W., Kampeera J., Arunrut N., Karuwan C., Sappat A., Khumwan P., Jaitrong S., Boonnak K., Prammananan T., Chaiprasert A. (2020). Graphene-based electrochemical genosensor incorporated loop-mediated isothermal amplification for rapid on-site detection of *Mycobacterium tuberculosis*. J. Pharm. Biomed. Anal..

[35] Aryan E., Makvandi M., Farajzadeh A., Huygen K., Bifani P., Mousavi S.-L., Fateh A., Jelodar A., Gouya M.-M., Romano M. (2010). A novel and more sensitive loop-mediated isothermal amplification assay targeting IS6110 for detection of *Mycobacterium tuberculosis* complex. Microbiol. Res..

[36] Iwamoto T., Sonobe T., Hayashi K. (2003). Loop-mediated isothermal amplification for direct detection of *Mycobacterium tuberculosis* complex, M. avium, and M. intracellulare in sputum samples. J. Clin. Microbiol..

[37] Yee E.H., Sikes H.D. (2020). Polymerization-based amplification for target-specific colorimetric detection of amplified I DNA on cellulose. ACS Sens..

[38] Divagar M., Bandaru R., Janakiraman V., Sai V.V.R. (2020). A plasmonic fiberoptic absorbance biosensor for mannose-capped lipoarabinomannan based tuberculosis diagnosis. Biosens. Bioelectron..

[39] Arshad N., Farooqi S.I. (2018). Cyclic voltammetric DNA binding investigations on some anticancer potential metal complexes: A review. Appl. Biochem. Biotechnol..

[40] Asrat T.M., Cho W., Liu F.A., Shapiro S.M., Bracht J.R., Zestos A.G. (2021). Direct detection of DNA and RNA on carbon fiber microelectrodes using fast-scan cyclic voltammetry. ACS Omega.

[41] Zamani M., Robson J.M., Fan A., Bono M.S., Furst A.L., Klapperich C.M. (2021). Electrochemical strategy for low-cost viral detection. ACS Cent. Sci..

[42] Ahmed M.U., Nahar S., Safavieh M., Zourob M. (2013). Real-time electrochemical detection of pathogen DNA using electrostatic interaction of a redox probe. Analyst.

[43] Dickinson E.J., Limon-Petersen J.G., Rees N.V., Compton R.G. (2009). How much supporting electrolyte is required to make a cyclic voltammetry experiment quantitatively “diffusional”? A theoretical and experimental investigation. J. Phys. Chem. C.

[44] Liu J., Tiefenauer L., Tian S., Nielsen P.E., Knoll W. (2006). PNA–DNA Hybridization Study Using Labeled Streptavidin by Voltammetry and Surface Plasmon Fluorescence Spectroscopy. Anal. Chem..

[45] Paleček E., Jelen F., Trnkova L. (1986). Cyclic voltammetry of DNA at a mercury electrode: An anodic peak specific for guanine. Gen. Physiol. Biophys..

