# Supplementary material for: Miniaturized Rapid Electrochemical Immunosensor Based on Screen Printed Carbon Electrodes for Mycobacterium tuberculosis Detection
Source: Biosensors (Basel). 2023 May 29;13(6):589. doi: 10.3390/bios13060589 (PMC10296126; doi:10.3390/bios13060589)
Supplement: Supplementary file 1 [file biosensors-13-00589-s001.zip › biosensors-2376688-supplementary.pdf]

Supplementary Information

Miniaturized Rapid Electrochemical Immunosensor Based on Screen Printed Carbon Electrodes for *Mycobacterium tuberculosis* Detection

Noura Zouaghi [1,2], Shahid Aziz [3,4], Imran Shah [5], Ahmed Aamouche [1], Dong-won Jung [6,*], Brahim Lakssir [2] and El Mostafa Ressami [2]

[1] LISA Laboratory, National Applied Science School, Cadi Ayyad University, Marrakech 40000, Morocco
[2] Moroccan Foundation for Advanced Science, Innovation and Research, Digitalization & Microelectronics Smart Devices Laboratory, Rabat Design Center, Rabat 10112, Morocco
[3] Department of Mechanical Engineering, Jeju National University, 102 Jejudaehak-ro, Jeju-Si 63243, Republic of Korea
[4] Institute of Basic Sciences, Jeju National University, 102 Jejudaehak-ro, Jeju-Si 63243, Republic of Korea
[5] Department of Aerospace Engineering, College of Aeronautical Engineering, National University of Sciences and Technology, Risalpur 24090, Pakistan
[6] Faculty of Applied Energy System, Major of Mechanical Engineering, Jeju National University, 102 Jejudaehak-ro, Jeju-Si 63243, Republic of Korea
* Correspondence: jdwcheju@jejunu.ac.kr

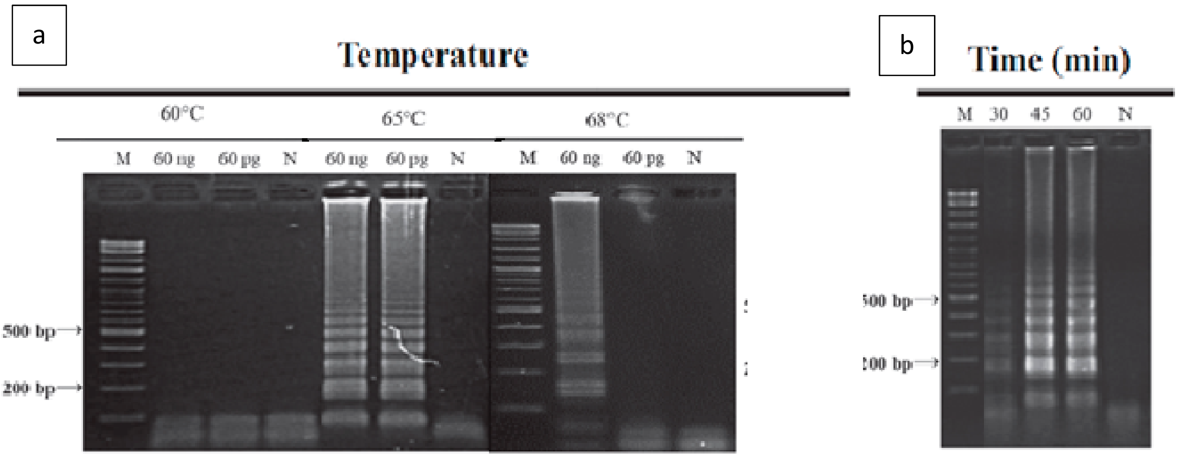

Figure S1. (**a**) Optimization of LAMP reaction temperature using 10-fold serially diluted Mtb-DNA as template. The reactions were carried out at different temperatures for 45 min.(**b**) Optimization of LAMP reaction time at the optimal temperature using various amounts of template.[1].

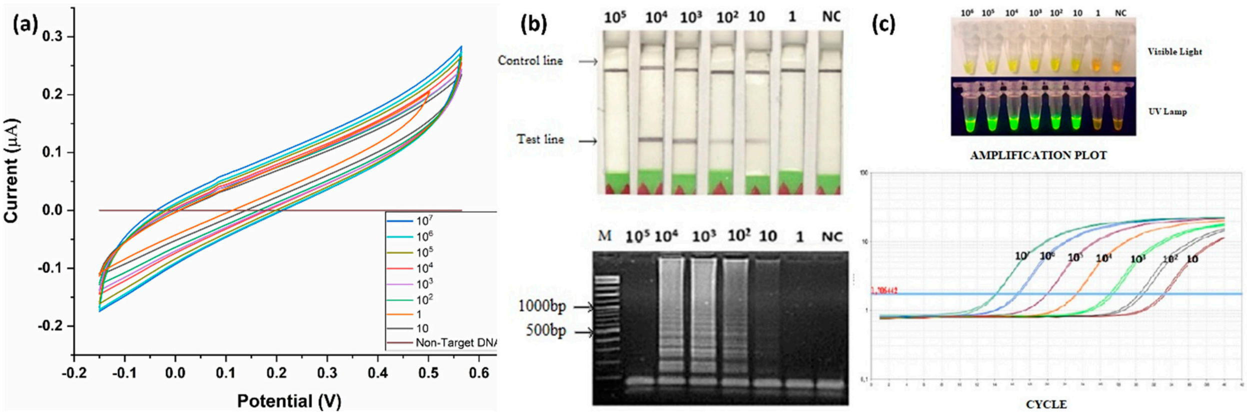

Figure S2. Detection sensitivity data of MTB genomic DNAs at concentration range of 10^{-1} to 10^{-6} dilutions obtained from (**a**) EC-LAMP, (**b**) LFD- LAMP, and (**c**) SS-LAMP. Lanes M and N represent DNA ladder marker and negative control (no-DNA template), respectively.

Table S1. Primer and probe sequences for the LAMP-EC biosensor-based assay.

Primer Name	IS6110 Gene Position	Sequence (5'-3')
F3 (FOP)	761-778	TCTCGTCCAGCGCCGCTT
B3 (BOP)	945-962	GCGGGTCCAGATGGCTTG
FIP (F1c-F2)	835-853/786-803	ACGTAGGCGAACCCTGCCCCC AGCACCTAACCGGCTG
BIP (B1c-B2)	861-879/922-939	GTCACCGACGCCTACGCTCTCG CGTCGAGGACCATGG
FLP	813-831	TCGACACATAGGTGAGGTC
BLP	901-918	TCGCTTCCACGATGGCCA

Table S2. Specificity of LAMP-EC using the mini-potentiostat.

Group of Bacteria	Species/Strain of Bacteria	TB Status by LAMP-EC
Tuberculous Mycobacteria	Mycobacterium tuberculosis	+
	Mycobacterium bovis	+
Non-Tuberculous Mycobacteria	Mycobacterium avium	-
	Mycobacterium fortuitum	-
	Mycobacterium intracellulare	-
Non-Mycobacterial Species	Bacillus cereus BCC 6386	-
	Bacillus subtilis BCC 6327	-
	Enterococcus faecalis	-
	Enterobacter aerogenes DMST 1333	-
	Escherichia coli O157:H7 ATCC 35150	-
	Listeria innocua DMST 9011	-
	Listeria welshimeri DMST 20559	-
	Pseudomonas aeruginosa	-
	Salmonella typhimurium ATCC 13311	-
	Salmonella enteritidis ATCC 13076	-
	Salmonella infantis DMST 26426	-
	Staphylococcus epidermidis TISTR 518	-
	Vibrio cholerae O1, DMST 22115	-
	Vibrio parahaemolyticus ATCC 17802	-

References

1. Jaroenram, W.; Kampeera, J.; Arunrut, N.; Karuwan, C.; Sappat, A.; Khumwan, P.; Jaitrong, S.; Boonnak, K.; Prammananan, T.; Chaiprasert, A.; et al. Graphene-Based Electrochemical Genosensor Incorporated Loop-Mediated Isothermal Amplification for Rapid on-Site Detection of Mycobacterium Tuberculosis. *J. Pharm. Biomed. Anal.* **2020**, *186*, 113333, doi:10.1016/j.jpba.2020.113333.
